# Persistent Staphylococcus aureus Isolates from Two Independent Cases of Bacteremia Display Increased Bacterial Fitness and Novel Immune Evasion Phenotypes

**DOI:** 10.1128/IAI.00255-15

**Published:** 2015-07-08

**Authors:** R. L. Richards, R. D. Haigh, B. Pascoe, S. K. Sheppard, F. Price, D. Jenkins, K. Rajakumar, J. A. Morrissey

**Affiliations:** a Department of Genetics and Infection, Immunity of Inflammation, University of Leicester, Leicester, United Kingdom; b Institute of Life Science, Swansea University College of Medicine, Swansea, United Kingdom; c Department of Zoology, University of Oxford, Oxford, United Kingdom; d Department of Clinical Microbiology, University Hospitals of Leicester, Leicester, United Kingdom

## Abstract

Staphylococcus aureus bacteremia cases are complicated by bacterial persistence and treatment failure despite the confirmed *in vitro* susceptibility of the infecting strain to administered antibiotics. A high incidence of methicillin-resistant S. aureus (MRSA) bacteremia cases are classified as persistent and are associated with poorer patient outcomes. It is still unclear how S. aureus evades the host immune system and resists antibiotic treatment for the prolonged duration of a persistent infection. In this study, the genetic changes and associated phenotypic traits specific to S. aureus persistent bacteremia were identified by comparing temporally dispersed isolates from persistent infections (persistent isolates) originating from two independent persistent S. aureus bacteremia cases with the initial infection isolates and with three resolved S. aureus bacteremia isolates from the same genetic background. Several novel traits were associated specifically with both independent sets of persistent S. aureus isolates compared to both the initial isolates and the isolates from resolved infections (resolved isolates). These traits included (i) increased growth under nutrient-poor conditions; (ii) increased tolerance of iron toxicity; (iii) higher expression of cell surface proteins involved in immune evasion and stress responses; and (iv) attenuated virulence in a Galleria mellonella larva infection model that was not associated with small-colony variation or metabolic dormancy such as had been seen previously. Whole-genome sequence analysis identified different single nucleotide mutations within the *mprF* genes of all the isolates with the adaptive persistence traits from both independent cases. Overall, our data indicate a novel role for MprF function during development of S. aureus persistence by increasing bacterial fitness and immune evasion.

## INTRODUCTION

*S*taphylococcus aureus is a commensal bacterium of human skin and mucosal membranes. Asymptomatic carriage is common, with as many as 80% of people colonized at any one time (1). However, this opportunistic pathogen is best known for causing a range of diseases from minor skin infections to bacteremia and septic shock. Infections can be classified as health care associated (HA) or community associated (CA), with the latter category being linked to the more virulent S. aureus strains (2, 3). Recorded incidences of methicillin-resistant S. aureus (MRSA) bacteremia in the United Kingdom have exhibited a decline in recent years, whereas the incidence of bacteremia caused by methicillin-susceptible S. aureus (MSSA) has remained relatively consistent (4, 5). However, both MRSA and MSSA infections remain a major problem worldwide (6). The problem is further exacerbated by the proliferation of strains with resistance or reduced susceptibility to last-line antimicrobial agents such as vancomycin (7, 8) and daptomycin and linezolid (9, 10).

Bloodstream infections and bacteremia caused by S. aureus are further complicated by the phenomenon of bacterial persistence and treatment failure despite the confirmed *in vitro* susceptibility of the infecting strain to the administered antibiotics. Persistence is traditionally defined as the “continuation or recurrence of the infection over a period in excess of 7 days” (11–14). Up to 38% of MRSA bacteremia cases can be classified as persistent and are associated with poorer patient outcomes (15–17). It is still unclear how S. aureus evades the host immune system for the prolonged duration of a persistent infection.

Previous studies have identified several characteristics associated with isolates from persistent infections (persistent isolates). For example, persistent isolates have shown elevated resistance to host defense peptides (HDP), enhanced biofilm formation, increased adhesion capabilities, and accessory gene regulator (*agr*) dysfunction (11–14). Furthermore, small-colony variants (SCV) are frequently implicated in persistence. These subpopulations can be metabolically dormant, elicit a reduced immune response during infections, and display heightened antibiotic resistance (18–22). However, our overall understanding of the mechanisms employed by S. aureus for the development of bacteremia persistence is still limited, and those mechanisms need further investigation.

In this study, our aim was to define the molecular mechanisms that lead to S. aureus persistence by investigating the genetic and phenotypic differences between temporally dispersed isolates originating from two persistent S. aureus bacteremia cases and by comparing these traits with those of three S. aureus bacteremia isolates from resolved infections (resolved isolates) from the same genetic background. This approach defines any genetic mutations and resultant phenotypic changes that are specific to S. aureus that cause persistent bacteremia and not bacteremia *per se*. Several novel traits were associated specifically with both independent sets of persistent S. aureus isolates compared to the initial and resolved isolates. These traits included (i) increased growth under nutrient-poor conditions; (ii) increased tolerance of iron toxicity; (iii) higher expression of cell surface proteins involved in immune evasion and stress responses; and (iv) attenuated virulence in a Galleria mellonella larva infection model that was not associated with small-colony variation or metabolic dormancy such as has been seen previously. Biofilm formation increased in persistent isolates during survival *in vivo*; however, total biofilm levels were equivalent to those seen with resolved isolates, indicating that biofilm formation is not a persistence-specific mechanism. Whole-genome sequence analysis identified different single nucleotide mutations within the *mprF* genes of persistent infection isolates from both the independent cases which correlated with the emergence of persistence-associated phenotypes. These data led to the implication of a specific gene, *mprF*, in persistence development in S. aureus bacteremia.

## MATERIALS AND METHODS

### Bacterial isolates.

MRSA isolates relating to staphylococcal bacteremia cases were identified and collected from the University Hospitals of Leicester (UHL) archive and stored in tryptic soy broth (TSB; BD Diagnostics Systems) with 20% (vol/vol) glycerol at −80°C. Unless otherwise stated, isolates were cultured on Luria agar (LA; Oxoid) at 37°C in air followed by Luria broth (LB; Oxoid) and were incubated with shaking at 37°C. For nutrient-restrictive conditions, 6% horse blood agar (HBA; Oxoid) was used and strains were cultured at 37°C in 5% CO_2_ followed by CRPMI medium (CRPMI medium is RPMI 1640 medium [Sigma-Aldrich] depleted of metal ions via treatment with 6% [wt/vol] Chelex 100 [Sigma-Aldrich] but with 10% [vol/vol] untreated RPMI 1640 medium reapplied to provide the minimum elements required for growth) (23) cultured statically at 37°C in 5% CO_2_.

### Antibiotic susceptibility.

MIC Evaluator strips (Oxoid) were used in accordance with CLSI guidelines to assess antibiotic sensitivities (24, 25). 2% NaCl was added to the medium for the oxacillin experiments according to standard practice. All readings were recorded after 24 h and confirmed at 48 h. Antibiotic sensitivity measurements were repeated 3 times; therefore, a 2-fold dilution range has been reported as recommended by the CLSI.

### Growth analysis.

Nutrient-restrictive growth analysis was conducted by subculturing isolates into 20 ml Chelex-treated RPMI medium (pH 7.0) to reach an optical density of 0.1 at 600 nm (OD_600_) from a fresh overnight culture and incubating statically at 37°C in 5% CO_2_ for 24 h. To test the effect that toxic iron levels had upon growth, 200 μM FeSO_4_ was added to duplicate CRPMI inocula prepared as described above. All cultures were inverted until equal suspensions were achieved, and 1-ml samples were analyzed at OD_600_ with a Jenway 6705 UV-light/visible-light (UV/Vis) spectrometer. Experiments were repeated independently three times, means and standard deviations (SD) were calculated, and a two-tailed Student *t* test determined significance.

### Quantitative hemolysis activity.

Bacterial exoproteins, including hemolysins, were extracted from overnight cultures by centrifugation. The supernatants were sterilized and concentrated using Amicon Ultra Centrifugal Concentrators (Millipore). A log_2_ serial dilution of the concentrated supernatant was made using phosphate-buffered saline (PBS) in a round-bottomed 96-well plate (Nunc). The negative lysis control did not contain any bacterial supernatant. A 4% concentration of sheep and rabbit blood was prepared by resuspending 400 μl of concentrated sheep and rabbit red blood cells (Oxoid) in 10 ml PBS. A 50-μl volume of 4% sheep blood–rabbit blood was added to all wells, and the plate was incubated at 37°C for 30 min. The terminal dilution where complete hemolysis still occurred was determined by eye and recorded for each sample. Experiments were repeated independently in triplicate to confirm reproducibility.

### DNase activity.

DNase activity was assessed by spotting 10 μl from each overnight culture onto DNase agar plates (Fluka BioChemika) followed by incubation at 37°C for 24 h. After incubation, DNase plates were flooded with 1 N HCl, precipitating polymerized DNA and leaving clear halos where the DNA was hydrolyzed by bacterial DNases. The radiuses of the clear halos surrounding the bacterial colonies were measured and recorded. Experiments were repeated independently in triplicate to confirm reproducibility.

### Non-covalently bound cell surface extracts and iTRAQ LC-MS.

Cell surface-associated protein extracts were prepared as described previously (23), except 16-h CRPMI (pH 7.0) cultures were used. Cells were harvested by centrifugation and the bacterial pellet weights calculated to normalize protein loading and to avoid bias from potential growth differences. The bacterial cell pellet (10 mg) was resuspended in 100 μl of 125 mM MOPS (morpholinepropanesulfonic acid; pH 7.0)–2% sodium dodecyl sulfate and boiled for 3 min followed by centrifugation and supernatant recovery. Samples were equalized with respect to protein concentration as determined by *A*_280_ values (using a Thermo Scientific 2000c NanoDrop instrument) and sent to the Protein Nucleic Acid Chemistry Laboratory (PNACL) proteomic facility at the University of Leicester. iTRAQ (isobaric tag for relative and absolute quantifications) (AB Sciex UK Ltd.) analysis was followed by liquid chromatography-mass spectrometry (LC-MS) for a maximum of eight individual samples. Data were analyzed using Scaffold 4 software (Proteome Software Inc.), which calculated *P* values from individual analysis of variance (ANOVA) tests. *P* values of <0.001 and <0.0001 showed that the results presented were statistically and significantly different under the compared conditions (https://proteome-software.wikispaces.com/file/view/looking-at-iTRAQ-data-in-scaffold-qplus).

### Biofilm formation assay.

Biofilm assays were conducted as previously documented (23). Briefly, this involved inoculating quadruplicate wells of a 96-well flat-bottomed microtiter plate (Nunc) with 200 μl of diluted bacterial cell suspension at an optical density at 600 nm (OD_600_) of 0.05 followed by static incubation for 24 h at 37°C. OD_600_ values were recorded at 0 and 24 h, using a BMG Labtech FLUOstar Omega plate reader to record potential growth differences. The wells were washed three times with sterile phosphate-buffered saline (PBS) and heat fixed at 60°C for 30 min. The wells were stained with 200 μl of 1% safranin for 30 min and washed twice with deionized water. Biofilms values were measured at OD_490_. Experiments were repeated independently three times, means and standard errors were calculated, and a two-tailed Student *t* test was performed.

### Adhesion, invasion, and intracellular persistence assay.

An H9C2 cell line was seeded and maintained in Dulbecco's modified Eagle medium (DMEM) GlutaMAX (Gibco) with 10% fetal bovine serum (FBS; PAA Laboratories), and the cells were grown to 100% confluence in 12-well tissue culture plates (Nunc) in preparation for invasion experiments. These were conducted using DMEM GlutaMAX with 1% FBS, and this was used to replace the previous growth media at least 30 min before the start of each experiment. For the bacterial inoculum, 10-ml LB overnight cultures were harvested and the bacterial pellets were washed three times in sterile PBS and resuspended in PBS to an OD_600_ of 1.0, equivalent to approximately 10^7^ CFU per 10 μl. Inoculum CFU levels were confirmed during each experiment via serial dilution and plating. A 10-μl volume of each culture was used to inoculate triplicate wells in three separate 12-well plates containing confluent H9C2 cells. The plates were incubated at 37°C in 5% CO_2_ for 2 h. For two of the three plates, the medium was replaced with DMEM GlutaMAX with 1% FBS, including 200 μg/ml gentamicin (Sigma-Aldrich), and the reaction mixture was returned to the incubator for a further 3 h (invasion assay) and a further 72 h (intracellular persistence assay). The final plate was taken for the next step without further medium replacement or incubations (adhesion assay). At each of these time points, medium was aspirated and wells were washed twice with PBS. The H9C2 cells were then lysed in 1 ml of 1% Triton X-100 (Sigma-Aldrich)–PBS for 10 min at room temperature, serial dilutions were made, and suspensions were plated onto nonselective media to calculate bacterial CFU levels.

### Galleria mellonella larva virulence model.

Galleria mellonella larvae from Live Foods Ltd. (United Kingdom) were stored at 4°C for up to 1 week. Prior to each experiment, larvae were left overnight at room temperature to acclimatize. Overnight bacterial cultures in 5 ml LB were centrifuged, and pellets were washed three times in sterile PBS and resuspended at an OD_600_ of 0.1, equivalent to 10^6^ CFU per 20 μl. Groups of 10 *Galleria* larvae were used for each infecting isolate. Larvae were injected with 20 μl of bacterial inoculum via their last proleg using microfine 1-ml insulin syringes (BD) with a stepper repetitive pipette (Tridak) and were incubated at 37°C. Fatalities were recorded every 24 h for 96 h. Groups of 10 larvae were injected with 20 μl sterile PBS, and another 10 were not injected at all; these were used as negative controls. If a total of two larvae from either or both of the two control groups died, the experimental results were discarded. Experiments were repeated independently three times; results were plotted on Kaplan-Meier survival graphs, and log-rank statistical analysis was conducted with the aid of GraphPad Prism 5 software. Equal inoculum sizes were confirmed by serial dilutions and CFU plating from the inoculum at the start of each experiment.

### CFU counts at 48 h postinfection and RNA isolation from Galleria mellonella larvae.

Relative bacterial burdens were investigated 48 h postinfection, and additional larvae were infected in parallel with each virulence experiment for this purpose. At 48 h postinfection, two living larvae per infecting strain per experiment were homogenized in 2 ml sterile PBS using zirconia/silica beads (Thistle Scientific, United Kingdom) (4 by 2.5 mm) in a minibead beater (Biospec Products, Inc.) at a high speed for 30 s. These were centrifuged at 2,500 rpm for 5 min at 4°C, and the liquid phase was retained and kept on ice. Serial dilutions were made and plated onto Staphylococcus-selective media (Mannitol salt agar; Oxoid), and CFU levels were measured after a minimum of 48 h of incubation at 37°C. The remaining liquid phase was centrifuged at full speed for 15 min, and the pellets were resuspended in 100 μl Tris-EDTA (TE) buffer containing 3 mg/ml lysozyme and 40 μg/ml lysostaphin (TE^LL^; Ambi Products LLC, USA) and incubated at 37°C for 15 min. A 900-μl volume of TRI reagent (Sigma-Aldrich) was then added, and samples were incubated for a further 5 min at room temperature. After the addition of 200 μl of chloroform, samples were subjected to vortex mixing for 15 s followed by 15 min of incubation at room temperature. Samples were centrifuged for 15 min at full speed at 4°C, and the upper aqueous phase was transferred to a fresh tube for RNA isolation. A 0.5-ml volume of isopropanol (IPA) was added to the upper aqueous phase, and the reaction mixture was inverted for mixing and incubated at room temperature for 10 min. Samples were then centrifuged at full speed for 8 min at 4°C to pellet the RNA. The RNA pellet was then washed with 1 ml 75% ethanol and centrifuged again at 10,500 rpm for 5 min at 4°C. The pellet was briefly air dried before being resuspended in 50 μl diethyl pyrocarbonate (DEPC)-treated water and stored at −80°C.

### Quantitative reverse transcription-PCR (qRT-PCR).

RNA samples were DNase treated using Turbo DNase (Ambion) according to the manufacturer's instructions, and the resulting samples were quantified using a Thermo Scientific 2000c NanoDrop instrument and normalized so that 12 μl contained 2 μg of RNA. Samples were stored at −80°C. Omniscript reverse transcription kits (Qiagen) were used to convert RNA to cDNA, using 12 μl (2 μg) of DNase-treated RNA; RNase inhibitor (Ambion) (10 U/μl) and random hexamers (Applied Biosystems) (10 μM) were also used as recommended but were not supplied within the kit. Samples were stored at −20°C for up to 1 week. Quantitative PCR (qPCR) was conducted using Fast SYBR green technology and an ABI 7500 machine. Briefly, this involved the use of a 20-μl reaction mixture containing 10 μl Fast SYBR green master mix (Applied Biosystems), 0.5 μl forward (ACGGTCTTGCTGTCACTTATT) and reverse (TACACATATGTTCTTCCCTAATAA) 16S rRNA 10 μM primer stocks (26), and the resulting cDNA (12 μl). Cycle conditions of 94°C for 20 s and 40 cycles of 95°C for 3 s and 60°C for 30 s were used, ending in a disassociation curve to confirm accurate primer binding and disassociation. Two technical repeats were completed per RNA sample. The resulting threshold cycle (*C_T_*) values were inverted and multiplied by 1,000 to make them manageable for comparisons.

### Whole-genome sequencing.

Genomic DNA (gDNA) was prepared using a Wizard Genomic DNA purification kit (Promega) according to the manufacturer's instructions, with an added lysis step involving pellet resuspension in 100 μl of 50 mM EDTA containing 200 μg/ml lysostaphin and incubation at 37°C for 30 min. A further cleanup step was used involving genomic DNA clean and concentrator columns (Cambridge Bioscience) according to the manufacturer's instructions. A minimum of 4 μg purified gDNA (minimum concentration, 40 ng/μl) resuspended in distilled water (dH_2_O) was submitted to the Swansea Genome Centre (Swansea, United Kingdom) and subjected to paired-end high-throughput genome sequencing on an Illumina HiSeq 2500 machine. Bioinformatic analysis was conducted using SPECTRE (Special Computational Teaching and Research Environment) at the University of Leicester. Raw paired-end reads were quality checked and trimmed using a FASTX toolkit. Trimmed reads were aligned to a reference genome (HO 5096 0412) using Picard, BWA-MEM, and SAMtools. Genetic variations compared to the reference genome and between the sequence data of individual isolates were identified using VarScan.v2.3.6 and a variant threshold of >80%. Additional sequence analysis involved the assembly of short reads into contiguous sequence files using Velvet software (27). These were entered into the BIGS Staphylococcal database, hosted at Swansea University, for gene-by-gene analysis (28). Open reading frames were identified using a reference pan-genome approach (29), and a core genome of 2,918 genes found in 90% or more of the 12 isolates was defined. A neighbor-joining phylogenetic tree was constructed from a core genome alignment of 2,596,723 bp shared by all isolates using MEGA v6 (30).

## RESULTS

### Clinical and genetic backgrounds of isolates from persistent and resolved MRSA bacteremia cases.

**FIG 1:**
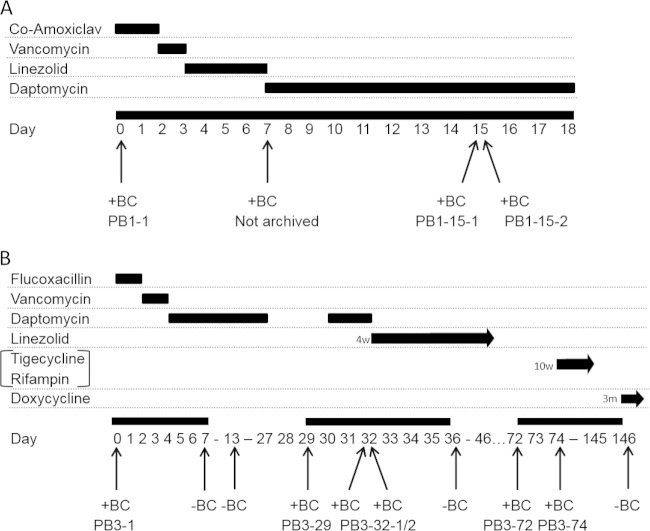
A diagrammatical representation of PB1 (A) and PB3 (B) treatment timelines. Time points for each blood culture collection and periods of antibiotic prescription are indicated relative to the initial positive blood culture for each bacteremia case. +BC, positive blood culture; −BC, negative blood culture; Co-Amoxiclav, amoxicillin-clavulanic acid.

**FIG 2:**
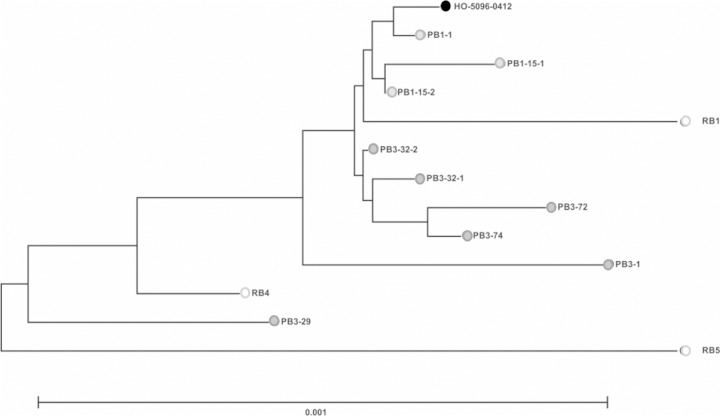
Genetic relatedness of bacteremia isolates. A neighbor-joining phylogenetic tree from a core genome alignment of 2,596,723 bp shared by all isolates constructed using MEGA v6 (30) is shown. The scale represents the number of substitutions per site. The reference EMRSA-15 genome (HO-5096-0412) is represented by a filled black circle, and resolved bacteremia isolates RB1, RB4, and RB5 are represented by open black circles.

To investigate the genetic and phenotypic traits specifically associated with S. aureus persistence, multiple temporal isolates were sampled from two patients admitted to the University Hospitals of Leicester between 2009 and 2010 who had been diagnosed with persistent MRSA bacteremia (infection cases designated PB1 and PB3). Three additional patients were diagnosed with resolved MRSA bacteremia (RB1, RB4, and RB5); i.e., the infections were treated successfully. Individual infection timelines were produced (Fig. 1), and relevant clinical information was recorded (Table 1). Two individual positive blood cultures were collected on day 15 and day 32, respectively, for both PB1 and PB3; this allowed the confirmation of homogenous blood-borne bacterial populations in those cases at those time points. PB1 was diagnosed with infective endocarditis following transthoracic echocardiography (TTE) on day 6, which offers a potential source for the persistent and, ultimately, fatal infection in this case. In contrast, the MRSA bacteremia in PB3 was diagnosed as a secondary infection associated with an ulcer in an infected foot, which was amputated on day 20, thereby eliminating the only identified focus of infection prior to the subsequent positive blood cultures (day 29 onward). On day 76 following the first positive blood culture, a spinal abscess and osteomyelitis were suspected, providing alternative possible infective foci in PB3. All MRSA bacteremia isolates, independently of infection type (persistent or resolved bacteremia), belonged to sequence type 22 (ST22) and *agr* type I and exhibited a staphylococcal cassette chromosome *mec* (SCCmec) type IVh element (31–34), indicating that these isolates were derivatives of Epidemic MRSA-15 (EMRSA-15). Whole-genome analysis revealed clustering of isolates as determined on the basis of homologous sequence variation at shared core genes (Fig. 2).

**TABLE 1:** Clinical details of MRSA persistent- and resolved-bacteremia cases used in this study^*a*^

Patient	Bacteremia classification	Antibiotic(s) prescribed (specific to MRSA BSI)	Positive MRSA BC(s)^*b*^	Days of negative BC collection post-initial culture	Suspected bacteremia focus and source seen at indicated time	Bacteremia outcome
PB1	Persistent	Vancomycin, linezolid, daptomycin	PB1-1, PB1-15-1, PB1-15-2	None	Day 6, TTE, mitral valve endocarditis	Patient died (day 18)
PB3	Persistent	Vancomycin, daptomycin, linezolid, tigecycline/rifampin, doxycycline	PB3-1, PB3-29, PB3-32-1, PB3-32-2, PB3-72, PB3-74	7, 13, 36, 37, 40, 42, 44, 47, 50, 57, 61, 146	Day 20, left foot ulcer (amputated); day 76, paraspinal abscess and right foot osteomyelitis	Patient discharged (day 146)
RB1	Resolved	Linezolid	RB1	NA	Infected right hemithorax and diaphragmatic patch	Resolved; patient discharged
RB4	Resolved	Vancomycin	RB4	NA	Long-term catheter (pyelonephritis) and JJ stents	Resolved; patient discharged
RB5	Resolved	Vancomycin	RB5	NA	Infected right prosthetic hip joint	Resolved; patient discharged

a BSI, bloodstream infection; BC, blood culture; PB, persistent bacteremia: RB, resolved bacteremia; JJ stent, ureteric stent; NA, not applicable.

b 1st number, bacteremia case number; 2nd number, day of BC collection; 3rd number, identification number of isolate from multiple BCs collected on the same day.

### Persistent isolates exhibited daptomycin nonsusceptibility.

All isolates were resistant to oxacillin and other β-lactams as confirmed by the oxacillin MICs being above the CLSI-recommended breakpoint (>2 μg/ml; Table 2). Data also showed that all EMRSA-15 PB3-derived isolates and all resolved bacteremia isolates were susceptible to vancomycin and linezolid (sensitivity, MIC ≤ 2 and ≤ 4 μg/ml, respectively). PB1 isolates showed intermediate resistance to vancomycin. Interestingly, daptomycin nonsusceptibility was judged to have developed during the PB1 and PB3 infections because the sequential isolates (PB1-15-1 and -15-2 and PB3-29, -32-1, and -32-2) showed a daptomycin resistance MIC (>1 μg/ml) whereas the cognate initial isolates (PB1-1 and PB3-1) showed susceptibility. Interestingly, PB3-72 and PB3-74 exhibited daptomycin susceptibility MIC values identical to that of the initial isolate (PB3-1).

**TABLE 2:** MICs for oxacillin, vancomycin, linezolid, and daptomycin^*a*^

Isolate	MIC (μg/ml)			
	Oxacillin	Vancomycin	Linezolid	Daptomycin
PB1-1	128	3–4	2	1
PB1-15-1	128	4	2	2–4
PB1-15-2	128	4	2	2–4
PB3-1	64	1–2	1–2	1
PB3-29	128	2	2	2–4
PB3-32-1	128	2	1–2	2
PB3-32-2	128	2	2	2–4
PB3-72	64–128	2	2	1
PB3-74	64	2	1–2	1
RB1	64	2	2	0.5
RB4	>256	2	2–4	0.5
RB5	64–128	2	1–2	0.5

a Experiments were repeated independently three times, and MIC data were recorded at up to a log_2_ dilution; a 2-fold dilution range is reported as recommended by the CLSI. MICs deemed to represent intermediate susceptibility or resistance according to published breakpoints are underlined. Resistance breakpoints (μg/ml): oxacillin, >2; vancomycin, >2; linezolid, >4; daptomycin, >1.

### Enhanced growth of persistent MRSA isolates in a nutrient-deprived growth environment and during iron stress.

**FIG 3:**
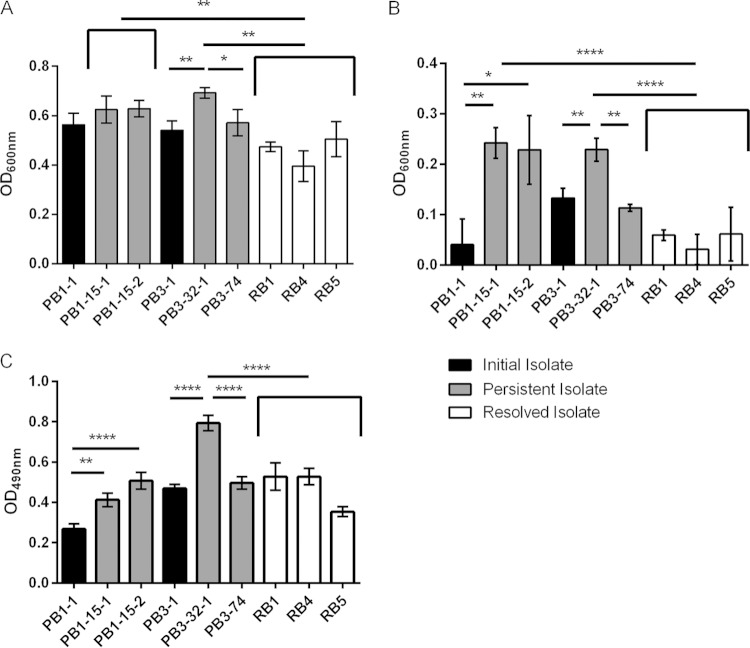
Growth analysis and biofilm assessment. (A and B) S. aureus strains were grown in iron-restricted nutrient-deprived CRPMI medium (A) and under conditions of iron stress in CRPMI medium with 200 μM FeSO_4_ (B). Growth was assessed by OD_600_ measurement after 24 h of static growth at 37°C in 5% CO_2_. (C) Biofilm formation was assessed by OD_490_ measurement after incubation in brain heart infusion (BHI)–1% glucose for 24 h. Data represent means and standard deviations of the results of at least three independent experiments. Significance is indicated with asterisks. *, *P* < 0.05; **, *P* < 0.01; ****, *P* ≤ 0.0001.

The formation of SCV has repeatedly been implicated in S. aureus persistence (18, 19, 21, 22); however, no significant differences in colony sizes between the initial, persistent, and resolved isolates examined in this study were observed, therefore indicating that none of the MRSA bacteremia isolates exhibited an SCV phenotype. Moreover, all MRSA bacteremia isolates showed no growth differences when cultured in a nutrient-replete medium (data not shown). In contrast, the EMRSA-15 PB1- and PB3-derived persistent isolates, PB1-15-1, PB1-15-2, and PB3-32-1, showed significantly increased growth compared to the control resolved EMRSA-15 bacteremia isolates when grown in nutrient-deprived, metal ion-restricted medium (Fig. 3A; *P* < 0.0001), a growth environment which is reflective of conditions *in vivo* (35). These data also showed that the persistent isolates had a growth advantage in CRPMI medium compared to the respective initial isolates (PB1-1 and PB3-1), although this difference was significant only for the PB3-1-versus-PB3-32-1 comparison (*P* < 0.01). Viability assays confirmed that persistent isolates showed an increased growth phenotype under nutrient-deprived conditions compared to both the initial infection and the control resolved isolates (data not shown). Interestingly, the measured growth of PB3-74 was equivalent to that of the initial PB3-1 isolate and was significantly lower than that of PB3-32-1 (*P* < 0.01), which is in agreement with the antibiotic resistance phenotypes.

Comprehensive assessment of the isolates' sensitivities was performed for a range of environmental stimuli. Interestingly, the EMRSA-15 PB1 and PB3 persistent isolates demonstrated significantly increased growth in the presence of toxic levels of iron (200 μM FeSO_4_) compared to the combined average of the growth measurements of the resolved isolates used as a control (Fig. 3B; *P* < 0.0001). In addition, persistent isolates PB1-15-1, PB1-15-2, and PB3-32-1 (Fig. 3B; *P* < 0.01 to 0.05) showed increased growth during iron stress compared to the respective initial isolates (PB1-1 and PB3-1). PB3-74 again displayed growth similar to that of the initial isolate (PB3-1), and the abundance of growth was significantly lower than that of PB3-32-1 (*P* < 0.01). These data suggest that the persistent isolates had acquired stable adaptations giving them a growth advantage under nutrient-deprived conditions and toxic iron stress conditions, both of which are environments that can be encountered during long-term survival *in vivo*; neither has been previously associated with S. aureus persistence.

### Persistent isolates show increased levels of cell surface-associated proteins involved in immune system evasion, host cell adhesion, and bacterial stress response.

**FIG 4:**
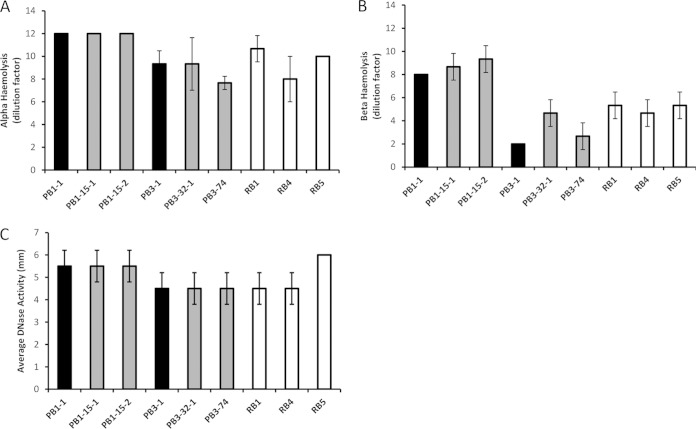
DNase and hemolysin activity. (A and B) Alpha (A) and beta (B) hemolytic activities were assessed as the final dilution (DF, dilution factor) of concentrated bacterial supernatant where complete hemolysis still occurred. Dilution factor means were calculated and plotted from the results of three independent experiments ± 1 standard deviation. (C) DNase activity was assessed by measuring the size (in millimeters) of the halo around the test isolates indicating hydrolyzed DNA. Means were calculated and plotted from the results of three independent experiments ± 1 standard deviation.

**FIG 5:**
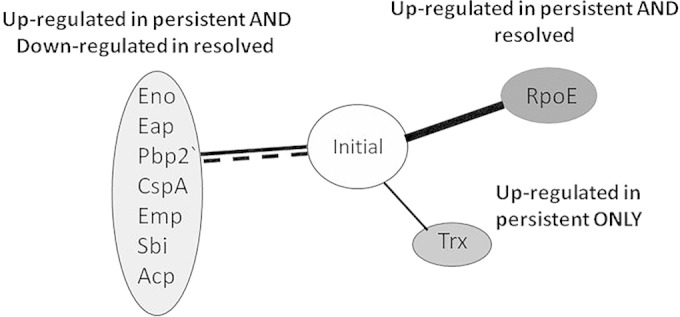
Summary of quantitative proteomic profiling of surface-associated proteins in EMRSA-15 persistent isolates and resolved-bacteremia EMRSA-15 isolates compared to the initial bacteremia isolates. Solid black lines indicate upregulation of proteins compared to the initial isolates; dashed lines indicate downregulation. The data representing the indicated proteins show changes relative to the initial isolates in one or more persistent or resolved isolate samples.

Persistent S. aureus bacteremia isolates have previously been associated with decreased toxin expression and activity, particularly of alpha-hemolysin, usually in combination with a recognized SCV phenotype and/or *agr* dysfunction (11, 13, 18, 19). No differences were found between the initial, persistent, and resolved MRSA bacteremia isolates with regard to alpha-hemolysin, beta-hemolysin, and DNase activity or cell wall covalently bound protein profiles in this study (Fig. 4 and unpublished data). However, several reproducible differences between the persistent, initial, and resolved EMRSA-15 bacteremia isolates in the non-covalently bound cell surface-associated protein fraction after growth in nutrient- and metal ion-restricted CRPMI medium were recorded. To identify individual proteins, quantitative proteomic profiling was conducted using iTRAQ (isobaric tag for relative and absolute quantification) coupled with LC-MS (liquid chromatography-mass spectrometry). The iTRAQ–LC-MS analysis identified 214 and 178 individual proteins in each biological repeat across the full set of isolates tested, and mean relative protein values were calculated. Those proteins which exhibited >0.5 log_2_-fold differences (*P* > 0.05) in abundance between each persistent or resolved MRSA bacteremia isolate and the respective initial isolate are listed in Tables 3 and 4, and the data are summarized in Fig. 5.

**TABLE 3:** Quantitative proteomic profiling of surface-associated proteins in PB1 persistent-bacteremia isolates and EMRSA-15 resolved-bacteremia isolates compared to the PB1 initial isolate^*a*^

Accession no.	Protein name	Gene	0.5 log_2_ ratio			
			PB1-15-1	PB1-15-2	RB1	RB5
Q5HEI2	Map adherence protein	*eap*	**0.80**	**0.7**	**−0.75**	**−1.05**
Q2FIK4	Extracellular matrixbinding protein	*emp*	0.05	0.35	**−0.65**	**−1.15**
Q2YVZ4	Immunoglobulin binding protein	*sbi*	0.05	0.30	**−0.65**	**−2.15**
Q2FH36	Cold shock protein	*cspA*	0.40	0.30	**−0.85**	**−0.95**
Q5HJW3	Penicillin binding protein 2	*pbp2*′	**0.70**	**0.85**	**−0.70**	**−1.65**
A7WZT2	Enolase	*eno*	**1.75**	**1.75**	−0.15	0.10
A7X1J8	Acyl carrier protein		**0.45**	**0.25**	**−0.60**	**−0.80**
Q2YUM5	RNA polymerase	*rpoE*	**3.15**	**2.7**	**0.85**	**1.1**
Q2FHT6	Thioredoxin	*trx*	**2.10**	**0.95**	−0.25	−0.15

a Bold values represent significant relative changes of 0.5 log_2_ ratio differences between the initial PB1-1 isolate and persistent PB1 infection isolates or resolved EMRSA-15 infection isolates. Values without a minus sign represent increases; values with a minus sign represent decreases. Relative protein change data represent averages of the results of two biological repeats (inclusive of two technical repeats). Proteins identified showed *P* values of <0.001 and <0.0001, calculated using the Scaffold Q+ proteomic software from individual ANOVA tests, showing the significance of the difference in protein abundance between two conditions.

**TABLE 4:** Quantitative proteomic profiling of surface-associated proteins in PB3 persistent bacteremia isolates and resolved bacteremia (RB) EMRSA-15 isolates compared to the PB3 initial isolate^*a*^

Accession no.	Protein name	Gene	0.5 log_2_ ratio			
			PB3-32-1	PB3-74	RB1	RB5
Q5HEI2	Map adherence protein	*eap*	**1.1**	−0.20	0.0	−0.3
Q2FIK4	Extracelllar matrix binding protein	*emp*	**1.4**	−0.15	−0.2	**−0.8**
Q2YVZ4	Immunoglobin binding protein	*sbi*	**0.7**	−0.10	0.55	**−0.95**
Q2FH36	Cold shock protein	*cspA*	**0.75**	0.10	−0.4	**−0.55**
Q5HJW3	Penicillin binding protein 2	*pbp2*′	**1.1**	0.05	−0.1	**−1.15**
A7WZT2	Enolase	*eno*	0.1	−0.15	**−0.70**	**−0.5**
A7X1J8	Acyl carrier protein		**0.8**	0.00	0.0	−0.25
Q2YUM5	RNA polymerase	*rpoE*	**0.95**	0.30	0.1	0.35
Q2FHT6	Thioredoxin	*trx*	**1.1**	0.00	−0.45	−0.45

a Bold values represent significant relative changes of 0.5 log_2_ ratio differences between the initial PB1-1 isolate and persistent PB1 infection isolates or resolved EMRSA-15 infection isolates. Values without a minus sign represent increases; values with a minus sign represent decreases. Relative protein change data represent averages of the results of two biological repeats (inclusive of two technical repeats). Proteins identified showed *P* values of <0.001 and <0.0001, calculated using the Scaffold Q+ proteomic software from individual ANOVA tests, showing the significance of the difference in protein abundance between two conditions.

Interestingly, the proteomic profiling showed that several non-covalently associated surface proteins displayed increased levels in persistent isolates and decreased levels in the resolved isolates compared to the initial persistent bacteremia isolates, suggesting that these proteins play important roles in S. aureus persistence. Proteins with increased levels in the persistent isolates compared to the initial isolates in both the PB1 and PB3 infections included extracellular adhesion protein (Eap), penicillin binding protein 2a (PBP2A′), and acyl carrier protein. Enolase (Eno) levels showed a substantial increase only in the PB1 persistent isolates, whereas extracellular matrix binding protein (Emp), bifunctional autolysin (Atl), immunoglobulin binding protein (Sbi), and cold shock protein A (CspA) showed increased levels in the PB3 infection.

Proteins showing a decreased level in the control resolved isolates compared to the PB1 initial isolate included Eap, Emp, Atl, Sbi, CspA, PBP2A′, and acyl carrier protein. Enolase was the only protein to show a significant level decrease in resolved isolates compared to the PB3 initial isolate (PB3-1). RNA polymerase RpoE showed increased levels in both persistent and resolved isolates compared to the initial isolates, whereas the thioredoxin (Trx) stress response protein level was increased in persistent isolates only. The PB3-74 isolate did not show any significant differences from the PB3 initial isolate. Overall, these data suggest the PB1- and PB3-infecting strains evolved during their individual infections, leading to similar changes in their cell surface protein profiles (Fig. 5). Additionally, the levels of the majority of these altered proteins were decreased in the resolved isolates, indicating the importance of these proteins for S. aureus persistence during bacteremia.

### Persistent isolates exhibited enhanced glucose-mediated biofilm formation.

Bacterial biofilms have been previously implicated as general mechanisms for immune system modulation and antibiotic tolerance (36–38) and therefore could be involved in persistence development. Biofilm formation was detected in the isolates after incubation in the presence of glucose (Fig. 3C). Interestingly, the EMRSA-15 persistent isolates PB1-15-1 (*P* < 0.01), PB1-15-2 (*P* < 0.0001), and PB3-32-1 (*P* < 0.0001) exhibited glucose-mediated biofilm levels that were significantly higher than those produced by the respective EMRSA-15 initial isolates. However, only PB3-32-1 showed a biofilm level significantly higher than that seen with the control EMRSA-15 resolved bacteremia isolates (*P* < 0.0001). Therefore, these data show that, although the persistent isolates had evolved during the infection to increase biofilm formation, there was no significant difference from the control resolved bacteremia isolates with respect to the level of biofilm, suggesting that biofilm formation is not an important aspect of persistence in these cases.

### Persistent isolates did not exhibit enhanced adhesion, invasion, or intracellular persistence.

**FIG 6:**
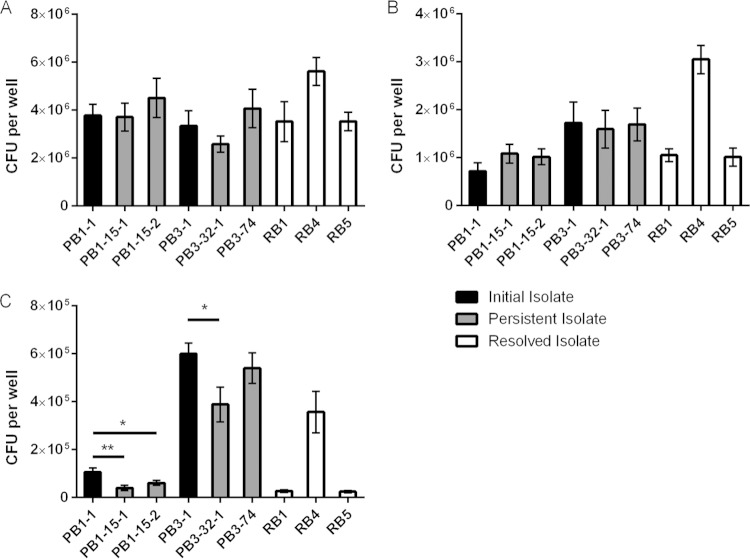
Adhesion, invasion, and intracellular eukaryotic cell persistence assay. Confluent H9C2 cells were cultured and infected with 10^7^ bacterial CFU, and the numbers of bacterial cells able to adhere (A), invade (B), and persist intracellularly (C) were counted. Significance is indicated with asterisks. *, *P* < 0.05; **, *P* < 0.01.

Host cell invasion and intracellular persistence have both been implicated as mechanisms of persistence (39, 40); therefore, we assessed these phenotypes using an H9C2 rat myocardium cell line infection assay (Fig. 6). No differences in adhesion or invasion rates were observed between the initial, persistent, and control resolved EMRSA-15 bacteremia isolates; in fact, resolved isolate RB4-1 showed a significantly higher invasion level than PB3-1, which was the isolate with the next highest invasion rate (*P* < 0.05). Furthermore, in contrast to previous studies, both the PB1- and PB3-associated persistent isolates actually showed reduced levels of intracellular persistence compared with the respective initial isolates (PB1-15-1, *P* < 0.01; PB1-15-2, *P* < 0.05; PB3-32-1, *P* < 0.05). These data show that there was no association between adhesion, invasion, or intracellular persistence capabilities and persistence in this study.

### Persistent isolates displayed attenuated virulence in a Galleria mellonella larva infection model.

**FIG 7:**
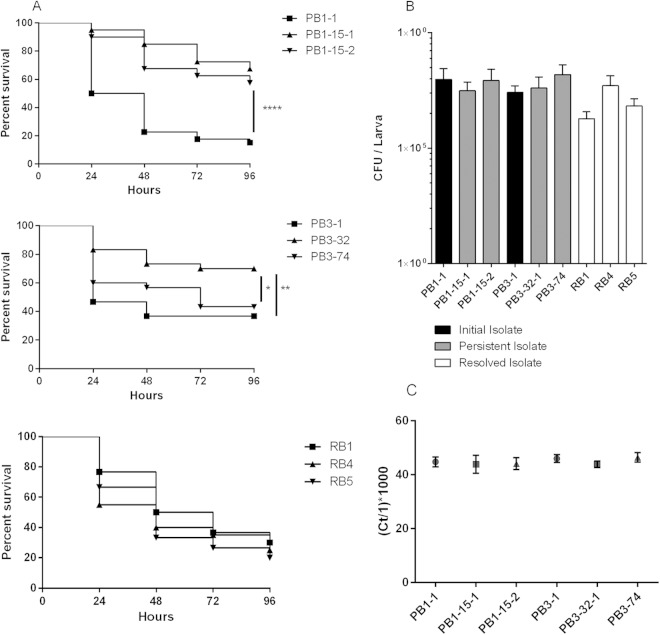
Galleria mellonella larva virulence assay. (A) Groups of 10 *Galleria* larvae were injected with ∼10^6^ CFU of bacteria and fatalities recorded every 24 h over a 96-h period. Data were plotted on Kaplan-Meier survival plots and log-rank tests used to determine significance. Significance is indicated with asterisks. *, *P* < 0.05; **, *P* < 0.01; ****, *P* < 0.0001. (B) Bacterial CFU levels were calculated from individual homogenized larva using selective media. (C) Total RNA was extracted from homogenized larvae, and 16S rRNA was quantified via qRT-PCR. *C_T_* values were inverted and multiplied by 1,000 to give manageable relative values for comparisons.

The general virulence behavior of the initial or persistent EMRSA-15 isolates was compared to that of the control resolved EMRSA-15 bacteremia isolates using a Galleria mellonella larva infection model where larvae mortalities are recorded every 24 h over a 96-h period (Fig. 7A). Persistent isolates associated with both PB1 and PB3 showed significantly lower virulence than the respective initial isolates (PB1-15-1, *P* < 0.001; PB1-15-2, *P* < 0.001; PB3-32, *P* < 0.01). There was also a significant difference between PB3-32-1 and PB3-74 (*P* < 0.05), with the latter exhibiting an enhanced *Galleria* killing phenotype equivalent to that seen with the PB3-1 initial isolate.

Intra-*Galleria* bacterial burdens were measured 48 h postinfection to assess the relative contribution of bacterial load to the virulence data previously observed (Fig. 7B). Importantly, none of the S. aureus colonies obtained following retrieval of bacteria from homogenized infected *Galleria* larvae exhibited a SCV phenotype. One-way analysis of variance (ANOVA) showed no significant differences in CFU per larva (CFU/larva) at 48 h between the *Galleria* larvae infected with initial or cognate persistent MRSA bacteremia isolates (PB1 isolates, *P* = 0.21; PB3 isolates, *P* = 0.27). However, the CFU/larva data differed substantially even between individual larvae infected with the same isolate within the same experiment (coefficient of variation, 95.72% to 228.69%). Therefore, an alternative, molecular quantification method was employed involving 16S rRNA quantitative reverse transcription-PCR (qRT-PCR) and RNA extracted from homogenized *Galleria* specimens (Fig. 7C). According to the data determined by this non-culture-based molecular method, intra-*Galleria* variation was substantially reduced but there were still no significant differences observed between the PB1- or PB3-associated initial isolates and the persistent isolates, as shown by one-way ANOVA (PB1-associated isolates, *P* = 0.14; PB3-associated isolates, *P* = 0.08). These data demonstrated that the attenuated virulence exhibited by persistent EMRSA-15 isolates derived from two individual bacteremia cases was not a result of differences in bacterial loads.

### All persistent isolates showing altered phenotypes had single nucleotide mutations in the *mprF* gene.

The reproducibility of the phenotypic traits associated with the persistent isolates in both cases of persistent EMRSA-15 bacteremia indicated that a stable, genetic basis for persistence had evolved during the two independent infections. Therefore, Illumina genome sequencing and whole-genome comparisons were conducted between the persistent and initial isolates within the individual infections to identify novel variants associated with persistence development.

FastQ files were quality checked, trimmed, and mapped to the closest available relative, EMRSA-15 (available from the Wellcome Trust Sanger Institute website; accession number HE681097 [HO 5096 0412]) (41), and variations present at >80% were recorded. Totals of 137 and 295 single nucleotide polymorphisms (SNPs) and insertions or deletions (indels) were identified in the initial PB1 and PB3 isolates, respectively, compared to the EMRSA-15 reference strain (Table 5). Comparisons of the genome sequences of the persistent isolates and the respective initial isolates showed that a series of genetic mutations (between 2 and 18 per isolate) had occurred in the persistent isolates during the course of the infection (Table 6).

**TABLE 5:** Summary of single nucleotide polymorphisms and insertions and deletions in infection isolates compared to the EMRSA-15 genome (HO 5096 0412)^*a*^

Isolate	No. of SNPs^*b*^	No. of indels	Total no. of SNPs and indels^*b*^	No. of SNPs and indels shared with respective initial isolate^*c*^
PB1	122	15	137	NA
PB1-15-1	124	15	139	136
PB1-15-2	119	14	133	132
PB3-1	273	23	296	NA
PB3-32-1	270	22	292	285
PB3-32-2	273	20	293	286
PB3-72	276	22	298	295
PB3-74	277	23	300	295

a SNPs, single nucleotide polymorphisms; indels, insertions and deletions.

b Data represent the number of SNPs and indels in each bacteremia isolate compared to the EMRSA-15 reference genome strain (accession number HE681097 [HO 5096 0412]) (42); variations present at >80% were recorded.

c Data represent the number of SNPs and indels identified from the EMRSA-15 reference strain that are shared between each of the persistent-bacteremia isolates and the corresponding initial isolate.

**TABLE 6:** Genetic changes associated with unique open reading frames in persistent-bacteremia isolates compared to the respective initial isolates

Isolate	Gene^*a*^	Position^*b*^	Reference nucleotide	Nucleotide variation(s)^*c*^	Function	Amino acid change or function
PB1-1	*SAEMRSA15_16780*	1860581	G	T	NAD binding	A271D
PB1-15-1	*mprF*	1343017	C	T	Membrane modification	P314L
	*pyrG*	2206316	G	A	CTP synthase	P412S
		2550433	T	C	Intergenic	
PB1-15-2	*mprF*	1344552	C	T	Membrane modification	L826F
PB3-29	*spa*	98739	G	A	IgG binding protein	D339E
		401923	T	−TTTAC	Intergenic	
		938796	G	T	Intergenic	
	*mprF*	1342960	C	T	Membrane modification	S295L
PB3-32-1	SAEMRSA15_00120*	16087	G	+T	Putative hydrolase	Promoter
	*spa*	98739	G	A	IgG binding protein	D339E
		401923	T	−TTTAC	Intergenic	
	SAEMRSA15_03710*	434768	T	G	Hypothetical	W1072L
		938796	G	T	Intergenic	
	*mprF*	1342960	C	T	Membrane modification	S295L
PB3-32-2	SAEMRSA15_00120*	16087	G	+T	Putative hydrolase	Promoter
	*spa*	98690	T	C	IgG binding protein	N356D
		938796	G	T	Intergenic	
	*mprF*	1344552	C	T	Membrane modification	L826F
	arsR2*	1861879	A	−ATAT	Arsenic resistance repressor	Promoter
	SAEMRSA15_18700*	2057502	A	T	Membrane protein	G2018G
	*fnbA*	2591495	T	C	Fibronectin binding	P892P
PB3-72	*spa*	98739	G	A	IgG binding protein	D339E
	SAEMRSA15_03710*	434757	T	+GGAAAAAATTG	Hypothetical	
PB3-74	*spa*	98739	G	A	IgG binding protein	D339E
		401923	T	−TTTAC	Intergenic	

a *, reversions to EMRSA-15 sequence from PB3-1 preexisting SNP.

b Data represent base positions in the EMRSA-15 reference genome sequence (accession number HE681097).

c Data represent variant bases relative to EMRSA-15 present at >80%.

The extensive pattern of shared SNPs seen in the genome analysis shows clearly that the persistent isolates had evolved from the respective initial isolates during the course of the infections. Interestingly, the mutation in the initial PB1 isolate in gene *SAEMRSA-15_16780* (which encodes a predicted NAD binding protein) that causes a nonpolar-to-acidic amino acid substitution (A271D) was repaired in both persistent PB1 isolates. Multiple other SNPs were also identified among the persistent isolates (Table 5); however, either these genotypic traits were found in only one of the persistent isolates from the infection case or the mutation was found to cause a conservative amino acid change. Two distinct mutations were identified in the *spa* gene in PB3 persistent isolates compared to the initial isolate; however, only one of the two mutations leads to a nonsynonymous amino acid change, and in all strains the *spa* gene appears to retain the frameshift mutation first identified in the EMRSA-15 genome sequence. The two different *spa* mutations do, however, indicate that PB3-29, PB3-32-1, PB3-72, and PB3-74 diverged from PB3-32-2 at an early stage of the infection, with PB3-29 and PB3-32-1 then gaining further mutations independently of PB3-72 and PB3-74.

Interestingly, the whole-genome sequencing revealed several different mutations in the *mprF* gene in the persistent isolates of PB1 and PB3 that were not observed in resolved EMRSA-15 isolates RB1, RB4, and RB5. The presence of different *mprF* SNPs, in separate divergent isolates taken from blood cultures on the same day, in two independent infections provides good evidence that there was a strong selective pressure for *mprF* mutation *in vivo* in these cases.

The terminal isolates of the PB3 lineage, PB3-72 and PB3-74, have the same synonymous SNP-containing *spa* alleles as PB3-29 and PB3-32-1; however, both PB3-72 and PB3-74 have the *mprF* allele of the initial isolate/wild type. This suggests that the *spa* mutation arose prior to the *mprF* mutation and that PB3-74 arose from a divergent bacterial population originating from the PB3 initial isolate prior to the emergence of PB3-29 and -32-1. In addition, the PB3-72 and PB3-74 isolates share 294 of the 295 SNPs and indels detected in PB3-1 (compared to the EMRSA-15 reference strain) whereas the PB3-32 isolates have only 285 of these original SNPs and indels, suggesting that the PB3-72 and PB3-74 isolates diverged directly from PB3-1. Therefore, the genome data support the phenotypic data showing that PB3-74 is more phenotypically similar to the initial PB3-1 isolate than to the other PB3 persistent isolates, such as PB3-32.

The sequence alignments of the initial and persistent isolates of both PB1 and PB3 were also examined using the Tablet viewer to look for large genomic insertions or deletions (43). Compared to the EMRSA-15 genome sequence, several large deletions or potential insertions were present in rRNA, phage, or transposon-related sequences of the bacteremia isolates; however, these mutations were found in all of the isolates, indicating that they likely had no role in the emergence of persistence (data not shown).

In summary, the *mprF* gene, in the two independent cases of persistent bacteremia, gained different mutations in the divergent persistent isolates that are strongly associated with the distinct novel phenotypic adaptations. These data therefore indicate that there is a strong association of *in vivo mprF* allelic variation with S. aureus persistence.

## DISCUSSION

In this study, we directly compared multiple temporal isolates from two independent cases of persistent EMRSA-15 bacteremia to contemporary isolates from resolved EMRSA-15 bacteremia infections. We identified several novel phenotypic traits which were associated exclusively with the persistent isolates from the two independent bacteremia cases and not with the respective initial isolate or the control resolved isolates. These novel persistence-associated traits include the following: (i) enhanced growth in a nutrient-deprived environment; (ii) increased tolerance of toxic iron conditions; (iii) increased abundance of cell surface-associated proteins involved in immune evasion, host cell adhesion, and bacterial stress response systems; and (iv) attenuated virulence in an insect larva infection model.

Whole-genome sequence comparisons identified several different SNPs in the *mprF* gene of the phenotypically adapted persistent isolates of both PB1 and PB3 bacteremia. The *mprF* SNPs were not found in non-phenotypically adapted persistent or resolved isolates, indicating that the *mprF* SNPs are associated with the genetic evolution of persistence. The three *mprF* SNPs identified in this study (S295L, P314L, and L826F) have previously been reported to be gain-of-function mutations and associated with the emergence of daptomycin nonsusceptibility and increased resistance to HDP (43–47). MprF is responsible for synthesis and translocation of lysyl-phosphotidylglycerol (L-PG) into the cell membrane, resulting in a reduced negative charge exhibited by the bacterial cell surface. It is proposed that enhanced MprF activity leads to greater abundance of L-PG in the cell membrane and an altered cell surface charge that leads to an increase in resistance to cationic antimicrobial agents such as HDP and calcium-coupled daptomycin (43–47). This is consistent with our observation of increased daptomycin resistance in the persistent isolates with *mprF* gain-of-function SNPs. However, the precise mechanism by which these *mprF* mutations lead to subsequent daptomycin nonsusceptibility remains unidentified. The fact that our study identified different causal *mprF* SNPs in separate isolates, taken from independent blood cultures collected on the same day, in two independent cases of daptomycin-treated persistent bacteremia provides significant evidence of a strong selective pressure for *mprF* mutation *in vivo*.

**FIG 8:**
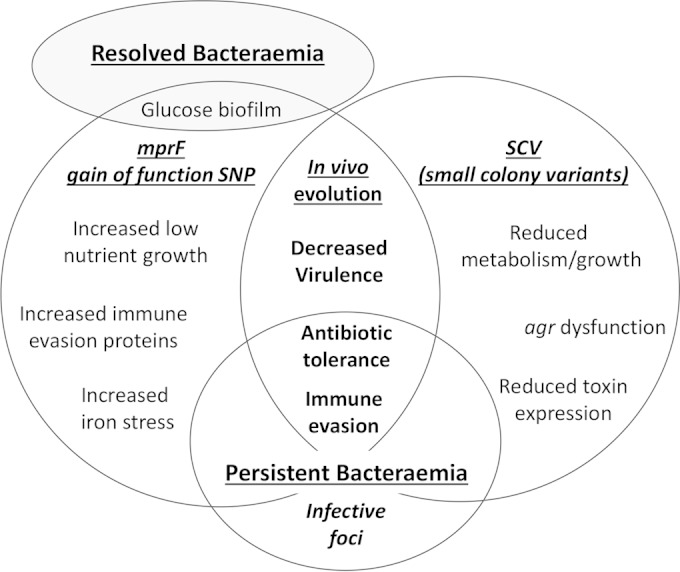
Model showing conserved mechanisms of S. aureus bacteremia persistence. Major mechanisms of persistence include *in vivo* genome evolution resulting in altered phenotypes and survival in a protected infective focus, both of which enable S. aureus to evade the immune system and tolerate treatment antibiotics.

Indeed, our data suggest that there are in fact multiple selective pressures *in vivo* because novel conserved phenotypic changes were identified in the isolates with *mprF* gain-of-function mutations. These *in vitro* phenotypic traits (detailed above) increase bacterial fitness *in vivo* by increasing growth and colonization under the nutrient-poor conditions found in a host and by promoting evasion of the immune system (Fig. 8). None of these phenotypic changes have been associated with persistence previously and so represent novel mechanisms of persistence.

Our data suggest that, in addition to *in vivo* genome evolution, another mechanism of persistence involves S. aureus “hiding” in a protected focus and then reemerging into the bloodstream. In the PB3 infection, the PB3-74 isolate showed antibiotic resistance, growth characteristics, and phenotypic traits identical to those seen with the initial PB3 isolate. In addition, the genome sequence analysis indicated that PB3-72 and PB3-74 share 100% of the EMRSA-15 reference genome SNPs and indels with the initial PB3-1 isolate, whereas PB3-32-1 and PB3-32-2 have only 96% identity, suggesting that the PB3-32 isolates have diverged further from PB3-1 than the PB3-72 and -74 isolates. Therefore, it is likely that the PB3-72 and -74 isolates reemerged into the bloodstream from a focus such as an abscess where the bacteria had been protected from the immune system and treatment antibiotics; they are unlikely to be growing at a high rate, hence the very low mutation rate.

Several persistence-associated traits have been proposed in previous studies. In this study, however, no evidence was found to support a correlation between SCV formation or eukaryotic intracellular persistence and persistent isolates. Indeed, SCV were not identified in any of the MRSA isolates used in this study, either during the initial clinical diagnosis at the University Hospitals of Leicester (UHL) or during subsequent *in vitro* analysis. This finding is significant because the formation of SCV is generally considered the main mechanism for persistence (18, 19, 21, 22). In addition, our study did not find any evidence of altered bacterial virulence, due to decreased exoprotein expression and/or reduced *in vivo* mortality, in combination with *agr* dysfunction and/or SCV formation as previously observed (11, 13, 18, 19). The persistent isolates in this study did, however, show attenuated virulence in an insect model of infection compared to the respective initial isolates or the resolved bacteremia isolates. Therefore, these data provide evidence for a role for reduced virulence in persistence that is not dependent on *agr* dysfunction, reductions in levels of secreted exoproteins or toxins, or SCV formation. Instead, our proteomic profiling found, for the first time, persistent isolate-specific increases in the levels of several non-covalently bound cell surface proteins.

The proteins with increased expression in persistent isolates included Emp and Eap, known mediators of low-iron biofilm formation, host protein adhesion enabling eukaryotic cell invasion (23, 48), and T-cell response disruption (49). The level of immune evasion protein Sbi, an immunoglobulin G binding protein, was also increased in persistent isolates (50, 51). The thioredoxin (Trx) and cold shock protein A (CspA) proteins and oxidative and cold shock pressure stress proteins also showed increased expression in persistent isolates (52, 53). Other proteins which showed differential abundance levels included PBP2′, enolase, a laminin adhesion protein (52), and two general cell maintenance proteins (acyl carrier protein and RNA polymerase subunit E [RpoE]).

Increased biofilm formation has previously been suggested to be a mechanism of persistence (14, 38). Consistent with this, our data suggest improved glucose-mediated biofilm formation in both the PB1- and PB3-infecting strains; however, the control resolved isolates demonstrated equally high levels of biofilms, suggesting that biofilm formation alone is not a specific persistence mechanism. This finding in particular emphasizes the importance of comparing initial infecting isolates with multiple persistent isolates from the same infection and contemporary resolved infection isolates to eliminate the possibility of bias introduced by any infection-specific traits.

Overall, our data suggest that the PB1- and PB3-infecting strains adapted to long-term survival within a host environment by upregulating surface protein-associated immune system defenses and stress response systems in combination with altered metabolism and virulence capabilities. Moreover, genomic evidence from independent infections shows a novel association between *mprF* mutation and the emergence of these novel persistence-specific virulence phenotypes during the progression of complex S. aureus bacteremia and the development of persistence *in vivo*. In summary, S. aureus has multiple mechanisms of persistence, including *in vivo* genome evolution and protection within a focus, which result in immune system evasion and tolerance of treatment antibiotics (Fig. 8).
